# Are risk estimates biased in follow-up studies of psychosocial factors with low base-line participation?

**DOI:** 10.1186/1471-2458-11-539

**Published:** 2011-07-08

**Authors:** Linda Kaerlev, Henrik A Kolstad, Åse Marie Hansen, Jane Frølund Thomsen, Anette Kærgaard, Reiner Rugulies, Sigurd Mikkelsen, Johan Hviid Andersen, Ole Mors, Matias B Grynderup, Jens Peter Bonde

**Affiliations:** 1 Danish Ramazzini Centre, Department of Occupational Medicine, Aarhus University Hospital and Regional Hospital Herning, Norrebrogade 44, 2C, 8000 Aarhus C, Denmark; 2 Research Unit of Clinical Epidemiology, University of Southern Denmark, Sdr. Boulevard 29, Entrance 101, 3rd floor, 5000 Odense C, Denmark; 3 Center for National Clinical Databases, South, Odense University Hospital, Sdr. Boulevard 29, Entrance 101, 3rd floor, 5000 Odense C, Denmark; 4 National Research Centre for the Work Environment, Lersø Parkallé 105, 2100 Copenhagen OE, Denmark; 5 Department of Occupational and Environmental Medicine, Bispebjerg Hospital, Bispebjerg Bakke 23, Entrance 33, 2400 Copenhagen NV, Denmark; 6 Centre for Psychiatric Research, Aarhus University Hospital, Risskov, Skovagervej 2, 8240 Risskov, Denmark

**Keywords:** cohort study, health survey, non-response, psychosocial distress, affective disorders

## Abstract

**Background:**

Low participation in population-based follow-up studies addressing psychosocial risk factors may cause biased estimation of health risk but the issue has seldom been examined. We compared risk estimates for selected health outcomes among respondents and the entire source population.

**Methods:**

In a Danish cohort study of associations between psychosocial characteristics of the work environment and mental health, the source population of public service workers comprised 10,036 employees in 502 work units of which 4,489 participated (participation rate 45%). Data on the psychosocial work environment were obtained for each work unit by calculating the average of the employee self-reports. The average values were assigned all employees and non-respondent at the work unit. Outcome data on sick leave and prescription of antidepressant medication during the follow-up period (1.4.2007-31.12.2008) was obtained by linkage to national registries.

**Results:**

Respondents differed at baseline from non-respondents by gender, age, employment status, sick leave and hospitalization for affective disorders. However, risk estimates for sick leave and prescription of antidepressant medication, during follow-up, based on the subset of participants, did only differ marginally from risk estimates based upon the entire population.

**Conclusions:**

We found no indications that low participation at baseline distorts the estimates of associations between the work unit level of psychosocial work environment and mental health outcomes during follow-up. These results may not be valid for other exposures or outcomes.

## Background

Participation in population-based studies addressing determinants of health outcomes have declined in past decades in several countries [1-3]. This is also the case for studies of psychosocial work factors. Low participation in cross-sectional studies obviously may cause selection bias if participation is related to rating of the psychosocial work environment and the outcome. In prospective follow-up studies, however, the risk of selection bias has been considered low because participation cannot depend on future outcomes. Although direct links between participation, psychosocial work environment and health outcomes are not an issue, participation might be related to gender, age, personality, occupational and social factors that may be related to exposure as well as later health outcomes [4]. Therefore the possibility of biased risk estimates in prospective follow-up studies with low participation rate cannot be ignored. So far only few studies have examined this issue [5-8]. Although reported findings in general are reassuring indicating that risk estimates among respondents do not differ substantially from risk estimates based upon the entire population including non-respondents, such findings can hardly be generalized. Low participation may have causes that differ from one setting to another. The objective of this paper is to examine bias by non-participation in a prospective cohort study addressing occupational stressors and mental health. We compared risk ratios for sick leave and prescription of anti-depressive medication redeemed at pharmacies according to established and potential determinants among respondents and the entire source population including non-respondents.

## Methods

### The PRISME study

The Danish PRISME cohort study was designed for purposes of prospective studies of job related psychosocial determinants of major depression and other common mental disorders [9]. The cohort was established in 2007 by recruiting 502 work units with 10,036 employees within large public service workplaces in a Danish county. A total of 4,489 employees returned a completed questionnaire on the psychosocial work environment and health (participation rate 45%). The workplaces included hospitals, schools, day care centres, social, technical and environmental services and administration. For 490 work units, we identified the work-unit leaders. These work units employed 10,009 workers that comprised the source population. After exclusion of 41 participants with incomplete information, 468 work units and 4,448 participants (including 263 leaders) remained. The number of employees in a work unit ranged from 1 to 124. Characteristics of the cohort are given in Table 1 and a detailed description is given in [9].

**Table 1:** Number of employees, work-units and questionnaire respondents at baseline

	Employees	Work-units	Respondents
1. Source data	10.036	502	4.489

2. Leader identified	10.009	490	4.483

3. As in point 2 as well as participants report job demands, skill discretion and decision latitude in questionnaire for the analyses with comparison between respondents (n = 4.448) and non-respondents (5.561) at baseline.	10.009	490	4.448

The PRISME study, Denmark, 2007.

### Measures of demographic characteristics

Information on gender, age, civic status and socio-economic position at study baseline in 2007 was obtained by linkage to public registries in Statistics Denmark. Civic status was defined as married or registered partnership versus all others. Socio-economic position was defined by level of education and current occupational position and classified into three categories: high, medium and low.

### Measures of the psychosocial work environment

The psychosocial working environment was characterized according to the demand-control [10], the effort reward imbalance [11] and the organizational justice [12] models and measured by established questionnaires.

Job demands, decision authority and skill discretion were each assessed by the mean score of four 5-level items, derived from the Copenhagen Psychosocial Questionnaire [13]. The mean score of the latter two scales was used to obtain a score for decision latitude. The combination of high job demands and low decision latitude using medians as level of dichotomies was defined as job strain. Effort-reward imbalance (ERI) was assessed as the ratio of the mean scores of three items addressing effort and the mean scores of seven items addressing reward [11]. Overcommitment at work, as measured by the intrinsic-effort dimension of the ERI model, was ignored in this study. Organizational justice was measured by the mean of item scores in the scales developed by Moorman [12]. Four items were used to assess procedural injustice and four items to assess relational injustice. In both scales responses were given on a five point Likert scale that ranged from strongly disagree to strongly agree.

The arithmetic mean values of each of the five scales of the psychosocial work environment were computed among employees by work-unit and this work-unit mean was subsequently assigned all non-respondents employed in the same work-unit. In the exposure-outcome analysis each of the scales was dichotomized into low and high (assigned) individual values by the median value.

### Measures of outcome

Using the unique personal identification code that all Danish citizens are assigned, we obtained complete information about sick leave exceeding two weeks and redeemed antidepressant prescription by linkage to the DREAM registry [14] and the Danish Medicinal Product Registry, respectively.

Data on sick leave were obtained from The National Register on Public Transfer Payments (DREAM), which contains weekly information on all public transfer payments for all residents in Denmark, and has been shown to be feasible for register-based follow-up of social and economic consequences of disease.

The Danish Medicinal Product Registry covers all pharmacies in Denmark. Antidepressants are only available by prescription in Denmark. The Medicinal Product Registry classifies prescribed Pharmaceuticals according to the Anatomical Therapeutic Chemical classification system (ATC) at the level of the generic pharmaceutical. We used prescription of one or more of the following drugs to define the endpoints for the present study: tricyclic antidepressants (TCA, ATC code N06AA), selective serotonin reuptake inhibitors (SSRI, ATC code N06AB), noradrenaline reuptake inhibitors (NARI, ATC code N06AX) and monoamin oxidase inhibitors (MAO-inibitors, ATC codes N06AF and N06AG). Lithium salts are mostly prescribed for bipolar affective disorders and were not included.

The study protocol was approved by The Danish National Committee on Biomedical Research Ethics, Region Central Denmark (RRS 2006-1028) and the Danish Data Protection Agency (2009-41-3215).

### Analysis

First, we compared respondents with non-respondents with respect to demographic characteristics. Second, associations between the average work-unit response rate and the average work-unit scores of the psychosocial work characteristics were examined by linear regression of the former on the latter, and in a multiple linear regression model adjusting for effects of all other psychosocial work factors. Third, we compared differences in risk estimates among respondents as compared with the entire study population: we analysed sick leave of more than 2 weeks (yes/no) and prescription of antidepressive medication redeemed at pharmacies at least once during the follow-up period from 1.5.2007 through 31.12.2008 by proportional hazard regression in the subsets of the study population that had no sick leave lasting 2 weeks or more from 1.1.2007-31.4.2007 (analyses of sick leave) or that had no prescription of antidepressive medication in the first four months of 2007 (analyses of antidepressive medication). For the comparison of differences in risk estimates among respondents as compared with the entire study population, we only included work-units with more than 5 employees, to avoid very unstable average measures of exposure. Time during follow-up was counted in weeks. Observations were censured if the participant died or emigrated during follow-up (n = 84). In analyses of sick leave, observations were also censured in case of retirement, rehabilitation or leave for other reasons except sick leave (e.g., education, pregnancy, child care). Hazard ratios (HR) were computed with respect to gender, age, civic status, social position and psychosocial work environment dimensions among questionnaire respondents and in the entire study population with adjustment for age and gender. Finally, we calculated relative risk ratios: defined as the risk estimate in the participant population divided by the risk estimate in the entire source population. Approximate confidence intervals were computed by the formula given in [8]. If the relative hazard ratio (RHR) is 1.0 there is no indication of bias.

## Results

Compared to non-responders, participants at baseline in 2007 were more often women [OR 1.84 (95% CI 1.8-2.2)], older than 45 years of age [OR 1.42 (95% CI 1.3-1.5)], more often of high social class [OR 1.53 (95% CI 1.4-1.7)] or medium social class [(1.93 (95% CI 1.7-2.2)], had less often sick leaves of more than 2 weeks duration [OR 0.84 (95% CI 0.8-0.9)], and were less often prescribed antidepressant medication in 2007, when the cohort was recruited.

**Figure 1:**
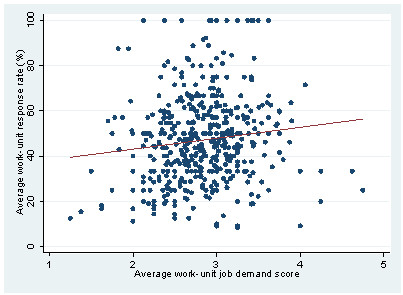
**The association between work-unit specific participation rate (%) and average scale scores of self-reported job demands**. The PRISME study, Denmark, 2007.

The association between the work-unit specific response rate and the average work unit scale score among questionnaire respondents are shown in Figure 1 for job demands. While the response rate increased with increasing job demands in a multiple linear regression model adjusting for effects of all other psychosocial work factors (p < 0.01), we observed no association for decision latitude. Likewise there was no association between work-unit response rate and the dimensions of the effort-reward imbalance model and the organisational justice models (data not shown).

The risk of sick leave and prescription of antidepressants during follow-up according to age, gender, civic status, socio-economic position and psychosocial work characteristics among responders and entire study population is provided in Table 2 and 3. When we calculated hazard ratios (HR), sixteen of 22 risk estimates differed by less than 10%, 19 by less than 15% and only the risk estimates for gender and decision latitude differed by more than 20%. None of the p-values for the relative HRs were less than 0.05. Risk estimates based on the subset of participants did only differ marginally from risk estimates based upon the entire population.

**Table 2:** Hazard ratio (HR) for use of antidepressants among respondents and for the entire study population

	Source population				Participant population				Relative risk estimates		
	**N**, **(%)**	Adj. HR	95% CI		**N**, **(%)**	Adj. HR	95% CI		RHR	95% CI	
Gender											
Men	91 (3.47)	1.00	Reference		22 (2.53)	1.00	reference				
Women	247 (3.90)	1.17	0.94	1.46	114 (3.61)	1.52	0.99	2.34	1.30	0.90	1.88
Age											
< 45	168 (3.54)	1.00	Reference		65 (3.36)	1.00	reference				
> 45	170 (4.03)	1.18	0.97	1.43	71 (3.39)	1.07	0.79	1.46	0.91	0.72	1.16
Civic status											
Living alone	151 (4.55)	1.00	Reference		60 (4.29)	1.00	reference				
Live together	187 (3.32)	0.65	0.53	0.79	76 (2.89)	0.61	0.45	0.84	0.95	0.74	1.21
Social status											
Low	72 (5.12)	1.00	Reference		23 (4.18)	1.00	reference				
Middle	151 (3.54)	0.69	0.54	0.90	73 (3.38)	0.81	0.53	1.26	1.17	0.83	1.66
High	63 (2.64)	0.52	0.38	0.71	28 (2.62)	0.62	0.37	1.05	1.20	0.79	1.82
Job demand at the group level											
Low	265 (3.70)	1.00	Reference		103 (3.22)	1.00	reference				
High	73 (4.08)	1.10	0.87	1.39	33 (3.99)	1.29	0.90	1.84	1.17	0.90	1.53
Decision latitude at the group level											
High	44 (3.48)	1.00	Reference		15 (2.61)	1.00	reference				
Low	294 (3.82)	1.12	0.84	1.50	121(3.50)	1.53	0.91	2.56	1.36	0.89	2.08
Jobstrain at the group level											
Low	286 (3.84)	1.00	Reference		116 (3.51)	1.00	reference				
High	52 (3.45)	0.89	0.68	1.16	20 (2.77)	0.77	0.50	1.21	0.87	0.62	1.24
ERI at group the level											
Low	241 (3.61)	1.00	Reference		96 (3.25)	1.00	reference				
High	97 (4.24)	1.25	1.01	1.54	40 (3.71)	1.22	0.87	1.70	0.98	0.76	1.26
Procedural injustice at the group level											
Low	272 (3.94)	1.00	Reference		111 (3.51)	1.00	reference				
High	66 (3.21)	0.91	0.72	1.16	25 (2.87)	0.92	0.63	1.35	1.01	0.75	1.37
Relational injustice at the group level											
Low	282 (3.95)	1.00	Reference		114 (3.53)	1.00	reference				
High	56 (3.07)	0.87	0.68	1.12	22 (2.76)	0.88	0.59	1.31	1.01	0.74	1.38

Total	338/8,958 (3,77)				136/4,028 (3,38)						

The PRISME study, Denmark 2007.

**Table 3:** Hazard ratio (HR) for sick leave above 2 weeks among respondents and for the entire study population

	Source population				Participant population				Relative risk estimates		
	**N**, **(%)**	**Adj. HR**	**95% CI**		**N**, **(%)**	**Adj. HR**	**95% CI**		**RHR**	**95% CI**	

Gender											
Men	349 (13.92)	1.00	reference		105 (12.47)	1.00	reference				
Women	1160 (19.40)	1.51	1.34	1.69	543 (18.11	1.55	1.26	1.90	1.03	0.87	1.21
Age											
< 45	792 (17.43)	1.00	reference		299 (16.13)	1.00	reference				
> 45	717 (18.18)	0.92	0.83	1.01	349 (17.57)	0.96	0.82	1.11	1.04	0.93	1.17
Civic status											
Living alone	692 (19.60)	1.00	reference		244 (18.53)	1.00	reference				
Live together	897 (16.73)	0.90	0.81	1.00	404 (16.01)	0.91	0.78	1.06	1.01	0.90	1.14
Social status											
Low	502 (23.57)	1.00	reference		180 (24.06)	1.00	reference				
Middle	723 (17.85)	0.80	0.70	0.92	349 (16.92)	0.72	0.59	0.89	0.90	0.77	1.05
High	284 (12.34)	0.58	0.49	0.68	119(11.56)	0.52	0.40	0.66	0.89	0.74	1.08
Job demand at the group level											
Low	1220 (17.99)	1.00	reference		522 (17.13)	1.00	reference				
High	289 (16.97)	0.94	0.82	1.07	126 (15.89)	0.96	0.80	1.16	1.03	0.89	1.18
Decision latitude at the group level											
High	196 (16.37)	1.00	reference		81 (14.7)	1.00	reference				
Low	1313 (18.01)	1.12	0.97	1.30	567 (17.24)	1.18	0.94	1.48	1.05	0.89	1.25
Jobstrain at the group level											
Low	1277 (18.10)	1.00	reference		552 (17.50)	1.00	reference				
High	232 (16.24)	0.87	0.76	0.99	96 (14.01)	0.78	0.63	0.96	0.90	0.76	1.06
ERI at group the level											
Low	1066 (17.12)	1.00	reference		458 (16.19)	1.00	reference				
High	423 (19.74)	1.14	1.02	1.27	190 (18.79)	1.14	0.96	1.34	1.00	0.89	1.14
Procedural injustice at the group level											
Low	1162 (17.74)	1.00	reference		513 (17.0)	1.00	reference				
High	347 (17.91)	1.05	0.94	1.19	135 (16.4)	1.00	0.83	1.20	0.95	0.82	1.09
Relational injustice at the group level											
Low	1180 (17.53)	1.00	reference		512 (16.58)	1.00	reference				
High	321 (18.79)	1.09	0.97	1.23	136 (18.09)	1.08	0.89	1.30	0.99	0.86	1.14

Total	1,509/8,486 (17.78)										

The PRISME study, Denmark 2007

## Discussion

The participation rate in the PRISME study was 45% and respondents differed from non-respondents at baseline by gender, age, social class and sick leave. The analyzed variables pointed to better health among participants. In spite of differential participation the study does not indicate that the association between the work place level of psychosocial work environment and mental health outcomes were systematically biased since risk estimates based on the subset of participants did only differ marginally from risk estimates based upon the entire population. These findings are in line with the results of few similar studies [4-8,15,16], but need cautious interpretation.

Limitations of self-reported measures of the psychosocial work environment have been acknowledged for many years [17,18]. As an alternative to crude objective indicators as overcrowding in hospital wards [19] and costly observational methods [20], the PRISME study relies on the average reporting of psychosocial work characteristics in managerial work-units. This approach assumes that psychosocial work characteristics are more homogeneous within than between work-units [9].

In studies of psychosocial work factors and health or sick leave, a slight overrepresentation of healthy, older, middle and high class women is expected, and may represent jobs with slightly different working conditions than the average. Furthermore, men and women may have different working conditions [21]. We have therefore adjusted all risk estimates between psychosocial factors and health for possible confounders: Age, gender, civic status, and social status.

Changing working conditions during a 20 months follow-up period may have played a role for the results. However, we expect that this may have been the case for both the risk estimates for the entire source population and the risk estimates for the subgroup of responders.

Our measure of work-unit specific psychosocial work characteristics were based upon the answers from those 45% that filled-in the questionnaire. When the work-unit average values were assigned to the non-respondents a bias could be introduced if the non-respondents perceived the working environment differently. In order to address this issue we analyzed the associations between the work-unit response rate and the average scale values for work characteristics among respondents. With one exception we found no such association indicating that biased estimation of exposure related to low response rate is unlikely. However, the perception of job demands was higher in work-units with high response rate. This might indicate that employees in work units with high job demands were more interested in participating in the study. If so, no reporting bias is expected when the rating of the respondents are assigned to the non-respondents within the same work-units. However, higher average job-demands in work units with high participation could also reflect a higher participation of employees that - everything equal - perceive the work as more demanding than other employees in the same department. Since the prescription of anti-depressive drugs was also higher among non-participants, bias of the exposure - outcome relation toward the null cannot be ruled out.

Although reassuring, findings for the limited number of outcomes that could be analyzed in both the subset of questionnaire respondents and the source population do not exclude biased risk estimates for other outcomes. On the other hand it is a strength that comprehensive measures of the psychosocial environment were available for the entire source population and that complete and independent information on the chosen outcomes was available.

## Conclusions

In conclusion, we found no indications that low participation distorts the estimates of associations between the work place level of psychosocial work environment and mental health outcomes.

## Competing interests

The authors declare that they have no competing interests.

## Authors' contributions

LK, HAK, ÅMH, JFT, AK, JPB coordinated the data collection in 2007. LK and JPB performed the statistical analysis for the present paper and drafted the manuscript. HAK, ÅMH, JFT, AK, RR, SM, JHA, OM, MBG have helped with interpretation of the analyses and with revising the manuscript critically. All authors participated in the design and coordination of the PRISME study, development of the questionnaire and have made substantial contributions to interpretation of data; and read and approved the final manuscript.

## Pre-publication history

The pre-publication history for this paper can be accessed here:

http://www.biomedcentral.com/1471-2458/11/539/prepub
